# The *HKT* Transporter Gene from *Arabidopsis*, *AtHKT1;1*, Is Dominantly Expressed in Shoot Vascular Tissue and Root Tips and Is Mild Salt Stress-Responsive

**DOI:** 10.3390/plants8070204

**Published:** 2019-07-04

**Authors:** Yuichi Tada

**Affiliations:** School of Bioscience and Biotechnology, Tokyo University of Technology, 1404-1 Katakura, Hachioji, Tokyo 192-0982, Japan; tadayui@stf.teu.ac.jp

**Keywords:** *AtHKT1;1*, promoter, reporter gene, salt-responsive, vascular tissue-specific expression

## Abstract

The *Arabidopsis* high-affinity K^+^ transporter (AtHKT1;1) plays roles in salt tolerance by unloading Na^+^ from the root xylem to the xylem parenchyma cells and/or uploading Na^+^ from the shoot/leaf xylem to the xylem parenchyma cells. To use this promoter for the molecular breeding of salt-tolerant plants, I evaluated the expression profile of the *AtHKT1;1* promoter in detail. Approximately 1.1 kbp of sequence upstream from the start codon of *AtHKT1;1* was polymerase chain reaction (PCR)-amplified, fused to the *β-glucuronidase* (*GUS*) gene, and introduced into *Arabidopsis*. The resultant transformants were evaluated under nonstressed and salt-stress conditions at the seedling and reproductive stages. Histochemical analysis showed that GUS activity was detected in vascular bundle tissue in roots, hypocotyls, petioles, leaves, and petals, and in root tips. GUS enzyme activity in shoots tended to be higher than that in roots at both stages. After treatment with 50 mM NaCl for 24 h, *GUS* transcription levels and GUS enzyme activity were enhanced in transgenic lines. These results indicate that the *AtHKT1;1* promoter isolated in this study could be useful in expressing transgenes specifically in vascular tissue and root tips, and in a mild salt-stress-responsive manner. The data provide novel insights into the functions of AtHKT1;1.

## 1. Introduction

Soil salinity is an environmental stress of major concern, causing significant loss of global agricultural productivity, particularly in irrigated soils [1,2]. Plant growth under salt stress requires the tight control of K^+^ and Na^+^ uptake, long-distance transport, and accumulation. Excess Na^+^ is toxic to the plant-cell cytosol and must be excluded from the cytoplasm [3]. Na^+^ enters roots passively, via nonselective cation channels and possibly other Na^+^ transporters, such as high-affinity K^+^ transporters (HKTs) [4]. HKTs, permeable either only to Na^+^ (class I) or to both K^+^ and Na^+^ (class II), are thought to play major roles in Na^+^ absorption and transport. In *Arabidopsis*, the HKT family comprises a single member, AtHKT1;1 (At4g10310), which is permeable only to Na^+^ [5,6]. However, reported physiological roles and the expression profile of AtHKT1;1 are controversial. Berthomieu et al. [7] concluded that AtHKT1;1 is involved in Na^+^ recirculation from shoots to roots by mediating Na^+^ loading into the phloem sap in shoots and unloading in roots. On the other hand, Davenport et al. [8], based on their experiments using radioactive tracers, concluded that AtHKT1;1 contributes to the retrieval of Na^+^ from the xylem, but is not involved in recirculation in the phloem. Sunarpi et al. [9] proposed that AtHKT1;1 contributes to Na^+^ removal from the ascending xylem sap and recirculation from the leaves to the roots via the phloem vasculature, based on the expression profile and analysis of AtHKT1;1 mutants. An et al. [10] confirmed with reciprocal grafting experiments between wild type (WT) and mutants that, in roots, AtHKT1;1 plays a role in *Arabidopsis* salt tolerance. Therefore, the major function of AtHKT1;1 remains to be determined in terms of salt tolerance in *Arabidopsis*: unloading Na^+^ from root xylem to xylem parenchyma cells, or uploading Na^+^ into phloem cells, followed by its recirculation to roots. The expression of *AtHKT1;1* has mainly been detected in roots [5], in the root stele, and vascular tissue in leaves [11], in the phloem of every organ [7], and in the xylem parenchyma and phloem cells in roots and shoots [9]. It was also reported that the expression of *AtHKT1;1* mRNA did not change in response to NaCl [5,7,10]; however, the *AtHKT1;1* gene was reported to be induced with 25–30 mM Na^+^ [9,12]. As the expression characteristics of a gene are closely related to the functions of the product, detailed analysis of the expression profile is important in elucidating function. In addition, the accurate characterization of the promoter is necessary if one wants to utilize this promoter to express transgenes in transgenic plants.

In this study, I analyzed spatial and developmental gene expression patterns, and responsiveness to moderate salt-stress of the *AtHKT1;1* promoter using a reporter gene. The results provide useful information on this promoter and reveal novel insights into the functions of AtHKT1;1.

## 2. Results

### 2.1. Production of Transgenic Arabidopsis Expressing β-glucuronidase (GUS) Gene Driven by AtHKT1;1 Promoter

The putative AtHKT1;1 promoter region, representing approximately 1.1 kbp of upstream sequence from the translation start site, was amplified by polymerase chain reaction (PCR) with specific primers, sequenced, and used to construct expression vector pAtHKT1–GUS. WT Arabidopsis plants (ecotype Columbia) were transformed with this expression vector by floral dipping, and 26 hygromycin-tolerant lines were obtained.

### 2.2. Histochemical Analysis of GUS Activity in Transgenic Lines

**Figure 1:**
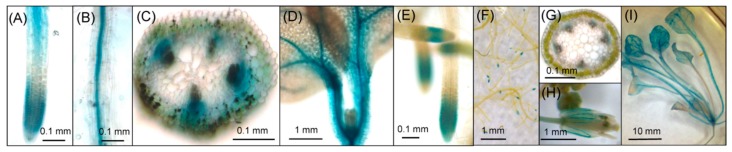
Histochemical analysis of *β-glucuronidase* (GUS) activity in typical T_2_ transgenic plants expressing the *AtHKT1;1pro*–*GUS* construct. Seven-day-old seedlings or plants at the reproductive stage (four weeks old) were used for GUS staining. (**A**–**D**) Plants at seedling stage; (**E**–**I**) plants at reproductive stage; (**A**, **E**) root tips; (**B**) middle part of the root; (**C**) hypocotyl cross-section; (**D**) cotyledon; (**F**) roots; (**G**) cross-section of the stem; (**H**) petals; (**I**) leaves. More than three different plants per line were analyzed, and photos of a representative plant are shown.

Fourteen independent T_2_ transgenic lines were histochemically analyzed by GUS staining using X-Gluc as the substrate (see Materials and Methods) at both the seedling and reproductive stages. At the seedling stage, strong GUS activity was detected in vascular bundle tissue of roots, leaves, leaf petioles, and hypocotyls, and in root tips in almost all examined lines (Figure 1, Table 1). At the reproductive stage, however, strong GUS activity was detected only in the vascular bundles of leaves, leaf petioles, and root tips, and faint GUS activity was detected in the vascular bundles of roots, stem, and petals in most lines (Figure 1, Table 1). GUS activity was very faint or not detected in the inflorescence and flower organs except for petal vascular tissue. Based on GUS staining patterns at the reproductive stage, transgenic lines could be categorized into four groups (Table 1): Group A, in which relatively strong GUS activity was detected in vascular bundles and root tips; Group B, in which GUS activity was not detected in vascular bundles of roots and stems; Group C, in which strong GUS activity was detected in vascular bundles of leaves, but weak activity in root tips and root vascular bundles; and Group D, in which GUS activity was strong in vascular bundles of leaf petioles, but faint in other types of tissue. Thus, GUS enzyme activity was specifically detected in vascular tissue and root tips, although there was a variability in activity among group transgenic lines.

### 2.3. Transcriptional Level of GUS Gene and GUS Enzyme Activity in Transformants

**Figure 2:**
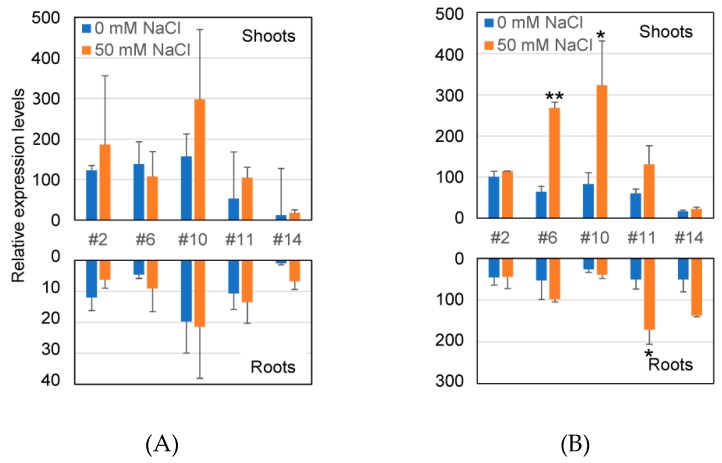
Transcriptional level of *GUS* gene in transgenic plants. (**A**) Seven-day-old seedlings; (**B**) plants at reproductive stage (four weeks old). RNA was extracted from the shoots and roots of transgenic plants grown on 1/2 MS agar medium, followed by incubation on 50 mM NaCl agar medium for 24 h and used for qRT-PCR. The expression levels of the ubiquitin extension protein were used for normalization of *GUS* expression. Expression levels are shown relative to that in the roots of Line 14 at the seedling stage under 0 mM NaCl (1.0). Data are presented the mean ± SE (*n* = 3, biological replicates). Single and double asterisks denote significant differences compared with values of plants at 0 mM NaCl treatment at *p* < 0.05 and *p* < 0.01, respectively, determined using Student’s *t*-test.

The transcriptional levels of the *GUS* gene under the *AtHKT1;1* promoter in transgenic lines were examined under nonstress and salt-stress conditions at both the seedling and reproductive stage by qRT-PCR analysis (Figure 2). In this study, five lines (2, 6, 10, 11, and 14) representing the four above groups (A–D) were used. After 24 h of salt treatment, a significant increase in *GUS* expression was observed in the shoots of Lines 6 and 10 and in the roots of Line 11 compared with the nonstressed samples at the reproductive stage (Figure 2B). *GUS* expression in other samples, except for Line 2, was also enhanced by salt treatment, although not significantly. At the seedling stage, the salt-responsive induction of *GUS* expression was not significantly detected (Figure 2A).

**Figure 3:**
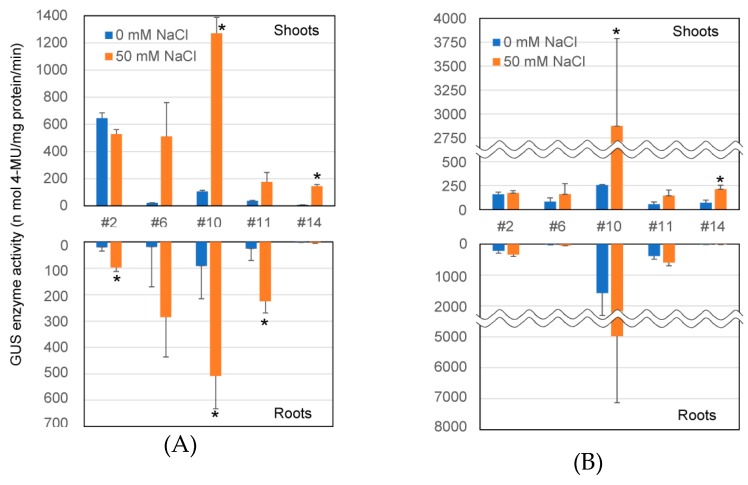
GUS enzyme activity in transgenic plants. (**A**) Seven-day-old seedlings; (**B**) plants at the reproductive stage (four weeks old). Protein was extracted from shoots and roots of transgenic plants grown on 1/2 MS agar medium, followed by incubation on 50 mM NaCl agar medium for 24 h and used for GUS enzyme assay. Fluorescence emitted by 4-MU, produced from 4-MUG by the GUS enzyme, was normalized per unit protein and per minute. Data are presented as mean ± SE (*n* = 3, biological replicates). Single and double asterisks denote significant differences compared with the values of plants at 0 mM NaCl treatment at *p* < 0.05 and *p* < 0.01, respectively, determined using Student’s *t*-test.

Salt-responsive GUS expression was also examined by GUS enzyme assay using 4-MUG as a substrate (Figure 3). Activity was significantly enhanced in both the shoots and roots of Line 10 by salt treatment at the seedling stage (Figure 3A). GUS enzyme activity in other samples, except for Line 2, tended to be enhanced by salt treatment at the seedling stage; however, this enhanced GUS activity was not apparent by the GUS staining assay (Figure S1A). Salt-responsive GUS expression in these lines was also observed at the reproductive stage (Figure 3B), and results were corroborated with the GUS staining assay (Figure S1B).

GUS activity in transgenic shoots tended to be higher than that in roots at both the seedling and reproductive stage, except for Line 11 at the seedling stage under salt stress and Lines 10 and 11 at the reproductive stage (Figure 3A,B). This dominant expression in the shoots was also observed by histochemical assay at the reproductive stage (Figure S1B).

### 2.4. In Silico Analysis of Regulatory Motifs in the Promoter Sequence

**Figure 4:**
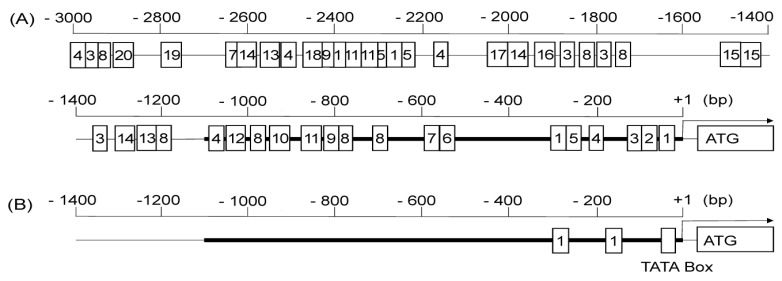
Schematic diagram of core promoter motifs predicted in the 5′ sequences of *AtHKT1;1*. The region of approximately 3 kbp upstream of the *AtHKT1;1* gene was searched for potential regulatory motifs. Possible cis-elements (motifs) were predicted by (**A**) the *Arabidopsis* Gene Regulatory Information Server [13] and (**B**) the plant-promoter database, version 3.0 [14]. The promoter region of approximately 1.1 kbp used in this study is shown in bold line. In (**B**), the region upstream of –1400 bp was omitted. Numbers in boxes indicate potential regulatory motifs; 1, ABRE-like binding-site motif; 2, SORLIP1; 3, GATA promoter motif; 4, T-box promoter motif; 5, G-box promoter motif; 6, SORLREP5; 7, ATB2/AtbZIP53/AtbZIP44/GBF5 BS in ProDH; 8, RAV1-A binding-site motif; 9, BoxII promoter motif; 10, AtMYC2 BS in RD22; 11, DPBF1 and 2 binding-site motif; and 12, GCC-box promoter motif; 13, LFY consensus binding-site motif; 14, MYB4 binding-site motif; 15, CArG promoter motif; 16, Ibox promoter motif; 17, MYB binding-site promoter; 18, Bellringer/replumless/pennywise BS1 IN AG; 19, Hexamer promoter motif; 20, CCA1 binding-site motif.

Approximately 3 kbp of the *AtHKT1;1* promoter sequence was analyzed using the *Arabidopsis* Gene Regulatory Information Server [13] and plant-promoter database, version 3.0 [14], to identify and characterize putative regulatory motifs present in the promoters (Figure 4). Sixteen putative motifs were found in the 1.1 kbp of sequence used in this study that belonged to 12 different types. Among them, high salt-responsive motifs, an ABRE-like binding-site motif and a G-box promoter motif, were identified. Although both types of software detected two ABRE-like binding-site motifs, only one was located at the same site and the other at different sites. The promoters contain a number of other putative cis-acting elements detected by the *Arabidopsis* Gene Regulatory Information Server (Figure 4A). Thirty additional putative motifs, including two ABRE-like binding-site motifs and two G-box promoter motifs, were found in the further 1.9 kbp upstream sequence not used in this study (Figure 4A). These motifs may also be responsible for the expression profile of endogenous *AtHKT1;1*.

## 3. Discussion

In this study, I examined the expression profile of *AtHKT1;1pro-GUS* in transgenic *Arabidopsis*. By histochemical assay of transgenic lines, GUS activity was detected in the vascular tissue of roots, leaves, petioles, hypocotyls, and petals in transgenic *Arabidopsis* (Figure 1), in agreement with previous reports [5,6,7,9,10,11]. In addition, GUS activity was detected in the root tips in all the transgenic lines examined in this study. Although the transgenic lines could be categorized into four groups based on the GUS staining patterns (Table 1), GUS enzyme activity was commonly detected in the vascular tissue and root tips in all groups. As all transformants showed GUS activity in their root tips, the *AtHKT1;1* promoter used in this study very likely works in the root tips.

I also examined the *GUS* transcriptional level and GUS enzyme activity in transgenic lines under nonstressed and salt-stress conditions at both the seedling and reproductive stage. Although it has been reported that the transcription of *AtHKT1;1* does not significantly change in response to 0, 25, 50, or 100 mM NaCl stress [5,7,10], the mild salt-stress-responsive expression of *AtHKT1;1* was reported in studies by Sunarpi et al. [9] and Wang et al. [12]. In this study, *GUS* gene expression was induced at both the transcriptional and enzymatic level under mild salt-stress, especially at the reproductive stage (Figure 2 and Figure 3). Some discrepancies, found between transcriptional activity and enzymatic activity, can be attributed to the fact that transplanting plants in solid salt medium may cause uneven stress on the roots and that transcriptional activity was net activity at the time of measurement, while enzymatic activity was the total activity of the accumulated protein so far. Although approximately 1.1 kbp of the promoter sequences was used for the assay of *AtHKT1;1pro–GUS* expression in this study, Berthomieu et al. [7] and Mäser et al. [11] used approximately 2.3 and 0.84 kbp of the promoter sequences, respectively. Baek et al. [15] reported that 5.2 kbp of the *AtHKT1;1* promoter sequence was a complete promoter controlling endogenous *AtHKT1;1* expression, and it contained an enhancer element and a putative methylation target at about 3.9 and 2.6 kbp upstream of the start codon, respectively. The lack of these elements may be attributed to the altered expression profile of the shorter promoters mentioned above. Although the length of the promoter sequences used in other studies is unclear, the difference in promoter length may explain the inconsistent tissue specificity and/or salt response of *AtHKT1;1pro–GUS* expression in the studies. Although two putative salt-responsive motifs were detected in the promoter region in this study (Figure 4), it is possible that these motifs might not be functional because these motifs are known to be high salt-responsive. It is also possible that unknown regulatory motifs that regulate salt-responsive expression might exist in the promoter sequence. However, these results showed that the 1.1 kbp *AtHKT1;1* promoter sequence can be used to specifically drive transgene expression in vascular tissue and in a mild salt-stress-inducible manner in transgenic plants.

GUS enzyme activity in transgenic shoots tended to be higher than that in roots at both the seedling and reproductive stage, with some exceptions (Figure 3). Using a ‘complete’ *AtHKT1;1* promoter, GUS staining was dominantly detected in leaves compared with roots in the transgenic plants [15]. Considering that total protein concentration in the shoots was much higher than in roots, GUS enzyme activity in shoots must be substantially higher than that in the roots. This shoot-dominant promoter activity was further confirmed by the histochemical GUS staining of transgenic plants, especially at the reproductive stage (Table 1, Figure S1). As gene-expression profile is related to protein roles in plant cells, such shoot-preferred *AtHKT1;1* promoter activity may be advantageous for Na^+^ upload into the phloem in the shoots over Na^+^ unload from the xylem in the roots.

## 4. Materials and Methods 

### 4.1. Production of Transgenic Arabidopsis

The promoter region of *AtHKT1;1* was amplified by PCR using specific primer pairs, AtHKT1proF1 (5′-AATAAGCTTTCCCTCGTCTCTACTCGTTCA-3′) and AtHKT1proR2 (5′-ACGACTAGTACTGATGATAGCGATTCCTGT-3′), in which restriction enzyme sites *HindIII* and *XbaI*, respectively, were created. The CaMV35S promoter of destination vector pGH1 [16] was excised with restriction enzymes *HindIII* and *XbaI*, and replaced with amplified 1.1 kbp of the *AtHKT1;1* promoter region, digested with *HindIII* and *XbaI*, to construct destination vector pAtHKT1. The entry vector, pENTR–GUS (Thermo Fisher Scientific K.K., Tokyo, Japan) was reacted with the LR enzyme with destination vector pAtHKT1 to construct pAtHKT1–GUS. WT *Arabidopsis* plants (ecotype Columbia) were transformed with this expression vector by floral dipping [17], followed by the selection of transgenic seedlings at 30 µg/mL hygromycin medium. Agrobacterium strain GV3101 was used for transformation. Hygromycin-tolerant T_2_ lines were used for the GUS and qRT-PCR assays.

### 4.2. Plant-Growth Conditions

Seeds of *Arabidopsis* were sown in 1/2 MS medium supplemented with 1% sucrose. The plants were grown at 23 °C under a 16/8 h light/dark cycle with approximately 60 µmol m^−2^ s^−1^ light intensity. For salt treatment, 7- or 28-day-old plants grown in 1/2 MS agar medium were transplanted to 1/2 MS agar medium, supplemented with or without 50 mM NaCl. Plant samples were harvested for RNA isolation or GUS assay after 24 h further cultivation.

### 4.3. Real-Time qRT-PCR

Harvested plant samples were split into shoots and roots, and frozen with liquid nitrogen. RNAiso Plus (Takarabio, Ohtsu, Japan) was used to extract total RNA, and real-time qRT-PCR was performed, as reported previously [16]. *GUS*-specific primers GUS8R (5′-TCGTGCACCATCAGCACGTTATCG-3′) and GUS9F (5′-GGCCAACAGTTCCTGATTAACCAC-3′) were used. The relative expression levels of the *GUS* for ubiquitin extension protein (*UBQ5*, AT3G62250.1) were calculated using the delta-delta Ct method.

### 4.4. GUS Assay

GUS activity was detected and quantified according to the method of Jefferson et al. [18]. Quantification of the fluorescence emitted by 4-MU, produced from 4-MUG by the GUS enzyme in the solution, was measured using microplate reader SpectraMax iD5 (Molecular Devices JAPAN, Tokyo, Japan). GUS activity in each sample was normalized per unit protein and per minute. Protein concentration in the crude extract was determined using a Bio-Rad Protein Assay (Bio-Rad, Hercules, CA, USA). For the histochemical assay, whole plants of transgenic lines were immersed in GUS reaction buffer (1 mM X-Gluc (5-bromo-4-chloro-3-indolyl-β-d-glucuronide), 100 mM phosphate buffer, pH 7.0, 0.1% v/v Triton X-100, 0.5 mM K_3_Fe(CN)_6_, and 0.5 mM K_4_Fe(CN)_6_, and vacuum-filtered for a few minutes to promote the penetration of the GUS reaction buffer. Plants were then incubated at 37 °C overnight. Chlorophyll was removed from the leaves by soaking in several baths of 70% v/v ethanol.

### 4.5. Promoter and Regulatory Element Resources

The plant-promoter database, version 3.0 (ppdb, http://ppdb.agr.gifu-u.ac.jp) [14], and the *Arabidopsis* Gene Regulatory Information Server (https://agris-knowledgebase.org/) [13] were used to retrieve information on the putative core promoter structure and putative regulatory element groups of the *AtHKT1;1* promoter, respectively.

## 5. Conclusions

I have presented evidence that the *AtHKT1;1* promoter, at least the sequence used in this study, is specifically active in the vascular bundle tissue and root tips in transgenic *Arabidopsis*. Promoter activity was found to be higher in the shoot vascular bundle than in the roots. Moreover, my data indicate that the expression was induced by mild salt-stress. These findings show the usefulness of this *AtHKT1;1* promoter for expressing transgenes specifically in vascular tissue and root tips under mild salt-stress conditions. Additionally, the expression profile of the *AtHKT1;1* promoter provide novel insights into the debate about the functions of AtHKT1;1 in response to salt-stress.

## Figures and Tables

**Table 1:** Expression profile of *AtHKT1;1pro–GUS* in transformants.

		Reproductive Growth Stage					Seedling Stage				
Group	Plant #	Root Tips	Root Vascular Bundle	Hypocotyl Vascular Bundle	Leaf Vascular Bundle	Leaf Petiole Vascular Bundle	Root Tips	Root Vascular Bundle	Hypocotyl Vascular Bundle	Leaf Vascular Bundle	Leaf Petiole Vascular Bundle
A	2, 3, 10, 13	++	++	+	+++	+++	+++	+++	+++	+++	+++
B	4, 11	++	−	−	+	+	+++	+++	−/+	+++	−/+++
C	1, 6, 7, 8, 9, 12	+	+	+	+/++	+++	+++	++/+++	+++	+++	+++
D	5, 14	−/+	−/+	+	+	+/++	+	−/+	+++	+	+++

−, no activity; +, weak activity; ++, strong activity; +++, very strong activity.

## Supplementary Materials

The following are available online at https://www.mdpi.com/2223-7747/8/7/204/s1. Figure S1: Histochemical analysis of GUS activity in *AtHKT1;1pro*–*GUS* transgenic lines under nonstress and salt-stress conditions.
