# Supplementary material for: The HKT Transporter Gene from Arabidopsis, AtHKT1;1, Is Dominantly Expressed in Shoot Vascular Tissue and Root Tips and Is Mild Salt Stress-Responsive
Source: Plants (Basel). 2019 Jul 4;8(7):204. doi: 10.3390/plants8070204 (PMC6681212; doi:10.3390/plants8070204)
Supplement: Supplementary file 1 [file plants-08-00204-s001.pdf]

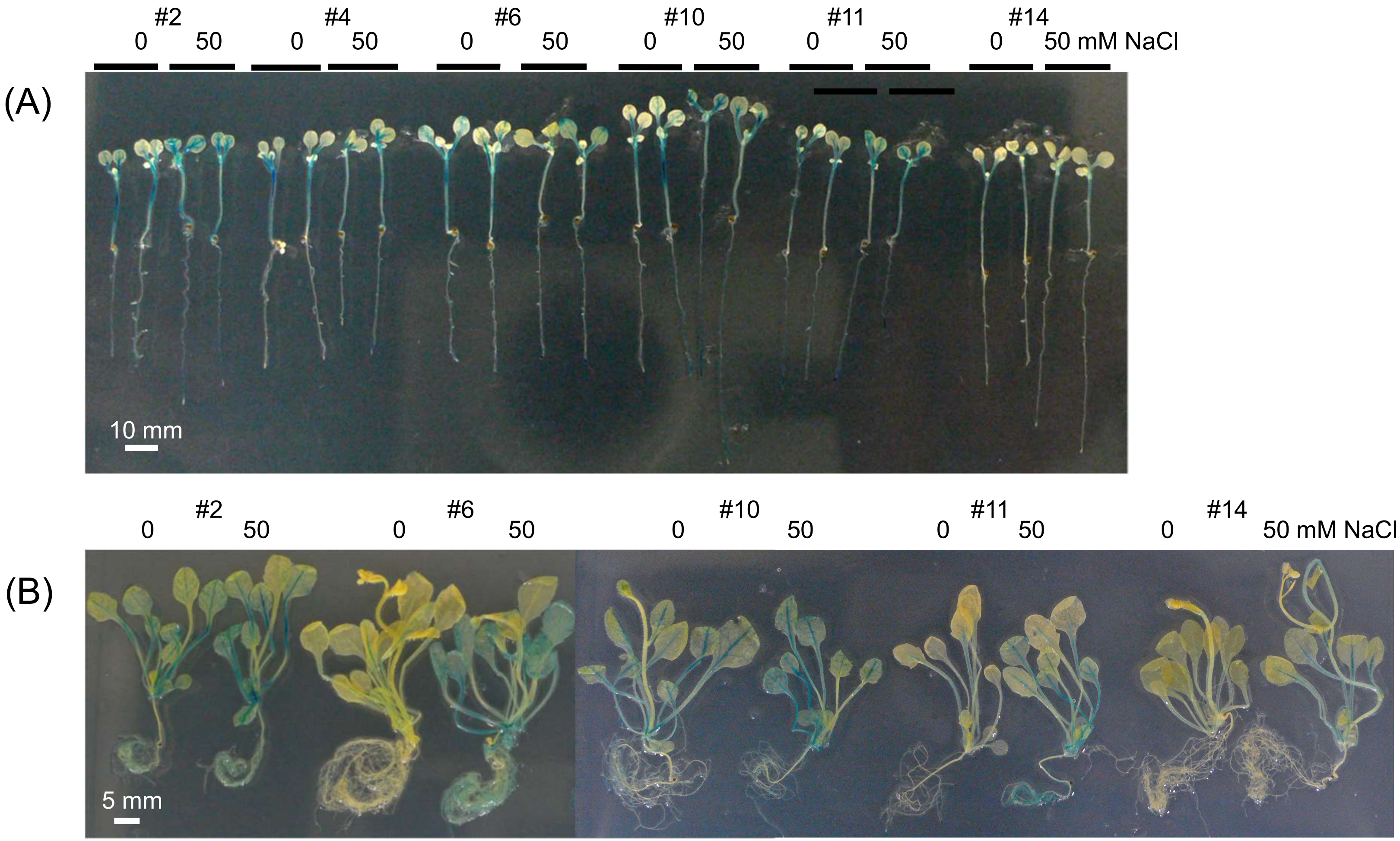

Figure S1: Histochemical analysis of GUS activity in *AtHKT1;1pro-GUS* transgenic lines under non-stress and salt stress conditions. (A) Seedling stage (7-day-old); (B) Reproductive stage (4-week-old). Plants were transferred onto 0 or 50 mM NaCl agar medium for 24 h and incubated with X-gluc.
